# Chinese Black Truffle-Associated Bacterial Communities of *Tuber indicum* From Different Geographical Regions With Nitrogen Fixing Bioactivity

**DOI:** 10.3389/fmicb.2019.02515

**Published:** 2019-11-05

**Authors:** Juan Chen, Jia-Mei Li, Yan-Jing Tang, Yong-Mei Xing, Peng Qiao, Yang Li, Pei-Gui Liu, Shun-Xing Guo

**Affiliations:** ^1^Key Laboratory of Bioactive Substances and Resources Utilization of Chinese Herbal Medicine, Ministry of Education, Institute of Medicinal Plant Development, Chinese Academy of Medical Sciences & Peking Union Medical College, Beijing, China; ^2^Shandong Institute of Sericulture, Shandong Academy of Agricultural Sciences, Yantai, China; ^3^Key Laboratory for Plant Diversity and Biogeography of East Asia, Kunming Institute of Botany, Chinese Academy of Sciences, Kunming, China

**Keywords:** Chinese black truffle, bacterial composition, geographical region, high-throughput, tissue culture, nitrogen fixation activity

## Abstract

It is well known that the microbes associated with truffle fruiting bodies play a very important role during the truffle lifecycle. *Tuber indicum*, commonly called Chinese black truffle, is a species endemic to Eastern Asia and in the genus of *Tuber*. Here, we reported the bacterial communities of *T. indicum* from different geographical regions and described the bacterial diversity from three compartments (soil, ectomycorrhizae and ascocarps) of *T. indicum* using high-throughput sequencing combined tissue culture. The results revealed that *Bradyrhizobium* was the dominant genus in fruiting bodies of *T. indicum* from nine geographical sites in China, and the microbes in *T. indicum* ascocarps were influenced by geological locations and soil characteristics. More specific bacterial taxa were enriched in the fruiting bodies than in the ectomycorrhizae and soil. In addition, 60 cultural bacteria were isolated from *T. indicum* fruiting bodies (4 families, 24 genera), and *Pseudomonas, Alcaligenes faecalis, Microbacterium*, and *Arthrobacter* were dominant. One of 13 strains that have potential nitrogen-fixation activities was further verified by an acetylene reduction assay (ARA). Together, this research provides new and important data for better understanding of the interaction between truffle and associated microbe and the biology of truffle itself.

## Introduction

*Tuber* is a genus belonging to Tuberaceae (Ascomycota) and usually produces hypogeous edible fruiting bodies, known as “true truffle” (Pegler et al., 1993). Some *Tuber* species have high economic value for their unique and delightful aromas, such as Italian white truffle (*T. magnatum*) and Perigord black truffle (*T. melanosporum*), which costing as much as 600€–6000€ per kilogram and are recognized as the most expensive foodstuffs in the world (Wang and Marcone, 2011; Torregiani et al., 2017). In addition, the summer truffle (*T. aestivum*) and the Chinese black truffle (*T. indicum* complex) are also very popular in the edible mushroom market. *Tuber* spp. can establish a tightly ectomycorrhizal symbiotic associations with Pinaceae (pines), Fagaceae (oak/beech), Myrtaceae (eucalyptus) and Salicaceae (willows/poplar) and benefit the growth of the host plants (Bonito and Smith, 2016); thus, *Tuber* spp. play an important role in nutrition recycling and utilization of forest ecosystems. Currently, truffles are cultivated in native habitats such as Spain, France, and Italy, as well as in non-native areas such as Australia and New Zealand by the mean of inoculating seedlings with spore suspension (Murat, 2015). Truffle cultivation involves environmental, economic, and social components that benefit tree species and forest ecosystems, providing high market value fruiting bodies as food and medicine and attracting interest from various social, ethnic, economic and age groups (Benucci et al., 2012). Moreover, as a means of land-use stability and reforestation and rural economic development, truffles are cultivated as long-term economic crops in some European countries such as France, Spain, and Italy (Bonet et al., 2006). Unfortunately, the yields harvested are low and highly variable due to climate factors, low genetic diversity, the quality of inoculums on nursery trees and the distribution of truffle mating types in truffle orchards (Linde and Selmes, 2012). In China, the natural production of truffle is gradually decreasing because of climate change (e.g., drought and low precipitation) and destruction of human activity (over-harvesting immature truffle), while artificial cultivation on a large scale is still not successful.

Previous studies on truffle evolution, species and genetic diversity, chemical composition and cultivation have made outstanding progress, but the biology of truffle fruiting bodies is still mysterious. It takes at least 5–7 years for truffle to complete a complex lifecycle from haploid spore germination to haploid free-living mycelium growth in soil, which includes the establishment of a mutualistic symbiotic association with roots of host plants and final formation and development of the fruiting body (Selosse et al., 2017). During the whole process, truffle interacts closely with microbes in the surrounding soil. Fluorescent *in situ* hybridization (FISH) confirmed the occurrence of dominant bacteria within mature ascocarps of *T. melanosporum* (Mello et al., 2013; Antony-Babu et al., 2014). Roux et al. (2016) suggested that bacteria of the genus *Rhodopseudomonas* (Bradyrhizobiaceae) establish obligate symbiosis with mycelia cultures of the black truffles *T. melanosporum*.

In past decades, microbial communities of several commercial truffle species have has been characterized with culture-dependent and 16S rRNA sequencing methods, including *T. borchii* (Barbieri et al., 2005), *T. aestivum* (Gryndler et al., 2013; Splivallo et al., 2019), *T. magnatum* (Barbieri et al., 2010; Leonardi et al., 2013; Amicucci et al., 2018), and *T. melanosporum* (Belfiori et al., 2012; Antony-Babu et al., 2013; Mello et al., 2013; Deveau et al., 2016; Roux et al., 2016). These studies demonstrated that truffle fruiting bodies harbor abundant and diverse microbial communities. Studies on the bacterial community of fruiting bodies of truffle indicated that *Pseudomonas*, *Bacteroides* and gram-positive bacteria were dominant in culturable bacterial communities (Sbrana et al., 2002; Barbieri et al., 2005), while Proteobacteria, Bacteroidetes, Firmicutes, and Actinobacteria formed the core component of the complex bacterial communities of all truffle species analyzed so far by culture-dependent and independent method techniques (Vahdatzadeh et al., 2015).

It has been reported that the bacterial community composition of truffle is potentially influenced by truffle species (Ye et al., 2018), geography (Benucci and Bonito, 2016), lifecycle stage (mycelium, ectomycorrhiza, and fruiting body) (Antony-Babu et al., 2014), host plant physiology (Li et al., 2018), seasons (Deveau et al., 2016), postharvest storage conditions (Vahdatzadeh et al., 2019), samples number and methodologies (Splivallo et al., 2019). Moreover, different tissues of fruiting bodies (e.g., peridium and gleba) were also enriched in specific bacteria (Antony-Babu et al., 2014). The microbial community associated with the truffle fruiting body has been deemed a complement of the external mycelium, which can promote the establishment of ectomycorrhizal symbiosis, modulate competition with other symbiotic and saprotrophic microbes and mobilize nutrients from minerals (Gryndler et al., 2013). Barbieri et al. (2010) demonstrated that nitrogen fixation occurs inside fruiting bodies of the white truffle *T. magnatum*, which may be related to presence of fixing bacteria associated with *T. magnatum* ascomata. Moreover, some microorganisms are believed to affect the production of the unique aroma of truffle (Buzzini et al., 2005). The unique aromas emitted from the fruiting body of truffle are responsible for its high economic value in the world market and determine the quality of the truffle. Early studies showed that yeasts isolated from the ascocarps of black and white truffles can produce volatile organic compounds (VOCs), speculating that these unique aromas might result from the interaction of truffle with their microbiome (Splivallo et al., 2011). Recently, it was demonstrated that bacteria isolated from truffle fruiting bodies were able to produce thiophene volatiles, which are sulfur-containing volatiles and characteristic of *T. borchii* fruiting bodies, suggesting that thiophene volatiles are exclusively synthesized by bacteria and not by the truffle extract during the sexual stage of truffles (Splivallo et al., 2015). Furthermore, correlations were detected between the changes in the volatile profiles of truffle and specific bacterial classes during the storage period of *T. aestivum* (Vahdatzadeh et al., 2019). These results highlighted the role of bacterial community as a most important contributor to the truffle aroma.

*Tuber indicum*, known as Chinese black truffle, is endemic to Eastern Asian and mainly distributes in China. The phylogenetic position of *T. indicum* belongs to the Melanosporum clade, which comprises species in Europe (*T. melanosporum* and *T. brumale*), Asia (*T. indicum* and *T. pseudoexcavatum*) and America (*T. regimontanum*) (Bonito et al., 2013). Among over 60 *Tuber* species of China, *T. indicum* is the most important species with the widest distribution and the largest production in China. Since the 1990s, *T. indicum* has been exported to the European market as the most primary trade wild edible mushroom of China. Due to the very close similarity with *T. melanosporum* in macro-and micro-morphological characteristics (black ascomata with conspicuous warts and spiny ascospores) and lower cost, *T. indicum* have attracted an increasing gastronomic and scientific interest in the past 20 years.

Previous studies on *T. indicum* have mainly focused on taxonomy (Liu and Tao, 1996), phylogeny (Wang et al., 2006; Zhang et al., 2010; Chen et al., 2011; Kinoshita et al., 2018), population genetics (Feng et al., 2016; Qiao et al., 2018) and mycorrhizal synthesis (Kinoshita et al., 2018). Only a few studies have attempted to explore the microbial community of *T. indicum* in the last 2 years. For example, Deng et al. (2018) investigated the bacterial community of *T. indicum* collected in Southwestern China using the traditional culture method and high-throughput sequencing and revealed that *Flavobacterium*, *Agromyces*, and *Ensifer* were dominant in fruiting bodies. Li et al. (2018) reported the microbial community structures of the ectomycorrhizosphere of *T. indicum* and host tree *Quercus aliena*. However, few studies have been carried out on the bacterial communities of *T. indicum* from different geographical regions systematically. Thus, the aims of this study are to compare the bacterial community of *T. indicum* from nine different geographical regions, to describe the bacterial community structure of ectomycorrhizae, fruiting bodies and soil in the same region using the high-throughput sequencing technique, to isolate the cultural bacteria from fruiting bodies and identify potential bacterial partners with biological activity. This study will provide useful information for understanding truffle development and realizing artificial cultivation of Chinese black truffles in the future.

## Materials and Methods

### Sample Collection

Fruiting bodies of *T. indicum* were collected from nine different geographical sites from October to November 2017. In addition, ectomycorrhizae and soil, and fruiting bodies, were collected from a *Castanea mollissima* orchard in Hebei Province. All samples were stored on ice during transport to the laboratory, after gently washed with sterile distilled water and then deposited at −80°C or used directly for subsequent morphological examination under a light microscope. A total of 120 fruiting bodies (FBs) of *T. indicum* were morphologically examined and classification of the maturation stage depends on the percentage of asci containing mature spores (Zeppa et al., 2002). Of them, fifty-four FBs were selected for bacterial community sequencing. Ectomycorrhizae of *T. indicum* were also collected under microscope according to the morphological characteristics (Geng et al., 2009). Rhizosphere soil adhering to the ectomycorrhizae was collected for microbial analysis and the bulk soils surrounding the fruiting bodies were collected for soil parameter analysis.

### DNA Extraction and Sequencing

For DNA extraction, 0.5 cm^3^ samples of healthy *T. indicum* fruiting bodies (no insect larvae galleries or animal injuries, etc.) from each geographical region were collected. Total DNA of ascocarps and ectomycorrhizae were extracted using a CTAB Plant DNA Extraction Kit (Aidlab Biotechnologies Co., Ltd., Beijing, China) according to the extraction protocol. Total DNA of soil adhering to the ascocarps (0.5–1 g) was extracted using the Power Soil DNA Isolation Kit (Mo Bio Laboratories, Carlsbad, CA, United States) according to the manufacturer’s protocol. DNA quality and quantity were assessed by the ratios of 260 nm/280 nm. For the 16S rRNA V3 + V4 region sequencing, samples from each region had 3–9 replicates. Amplification libraries were generated using the universal primers 338F (5′-ACTCCTACGGGAGGCAGCA-3′) and 806R (5′-GGACTACHVGGGTWTCTAAT-3′) combined with adapter sequences and barcode sequences (Mori et al., 2014). High-throughput sequencing analysis of bacterial rRNA genes was performed on the purified, pooled sample using the Illumina HiSeq 2500 platform (2 × 250 paired ends) at Biomarker Technologies Corporation, Beijing, China.

### Bacterial Community Analysis

The raw paired-end reads from the original DNA fragments were filtered and merged using FLASH v1.2.11 (Tanja and Salzberg, 2011), and the sequences were quality evaluated with PRINSEQ v0.20.4 (Schmieder and Edwards, 2011). High-quality reads from each sample were clustered into operational taxonomic units (OTUs) based on a 97% sequence similarity according to UCLUST (Version 1.2.22) (Edgar, 2010). Taxonomic assignments were performed against the RDP 16S rDNA sequence reference database with the naïve Bayesian RDP classifier and a minimum confidence level of 0.8 (Wang et al., 2007).

For alpha diversity analysis, we rarefied the OTUs to several metrics, including rare faction curve and Shannon curve (Wang et al., 2012), as well as rank abundance curve (Kõljalg et al., 2013). Shannon index (Rodrigues et al., 2014), Simpson index (Simpson, 1949), ACE index (Hughes et al., 2001) and Chao1 index (Chao et al., 2005) were calculated and plotted in the R statistical environment with the package phyloseq (Mcmurdie and Susan, 2013). For beta diversity analysis, a heatmap of RDA-identified key OTUs, principal component analysis (PCA) and Linear discriminate analysis effect size (LEfSe) analyses were performed for the quantitative analysis of biomarkers among each group. Briefly, for LEfSe analysis with an LDA threshold of >4, the non-parametric factorial Kruskal-Wallis (KW) sum-rank test was used, and the (unpaired) Wilcoxon rank-sum test was used to identify the most differently abundant taxa.

### Statistical Analysis

The effects of the geographic sites on the structures of the bacterial communities were determined by ANOVA at a threshold level of *P* = 0.05 and Fisher’s test. All statistical analyses were performed with SPSS (Version 23.0.0.0). Permutational multivariate analysis of variance (Anderson, 2010) was implemented in the R package vegan (Oksanen et al., 2019), and adonis and beta dispersion functions were used to test the null hypothesis that there are no differences among bacterial communities of *Tuber* species.

### Isolation and Identification of Cultural Bacteria

Ascocarp samples were briefly flamed, and approximately 1 g of gleba tissue was cut from the ascocarps using a sterile scalpel, and the sample was homogenized in 10 mL of filter-sterilized physiologic solution (0.85% NaCl) before shaking the flasks at 160 rpm for 2 h. Serial dilutions were prepared from 10^–2^ to 10^–6^, and 100 μL aliquots (10^–4^ to 10^–6^) were chosen to spread on Tryptone Soya Agar (TSA), and the plates were inoculated at 28°C to isolate the culturable bacteria. Three replications were carried out for each concentration. One hundred microliters of sterile water was used as a blank control. The plates were incubated for 2–5 days at 28°C. Morphologically different colonies that appeared in the medium were isolated and purified on plates containing TSA medium. The isolated strains were routinely subcultured every 4 weeks and stored in TSA medium amended with 25% glycerol at −80°C (Navarroródenas et al., 2016). The physiological and biochemical characteristics of the bacterial isolates were examined according to the methods described in Bergey’s Manual of Determinative Bacteriology Eighth Edition (Balows, 1975), and the bacterial isolates were also identified based on 16S rDNA sequencing. Bacterial DNA was extracted with a Genomic DNA Extraction Kit (TIANGEN, Beijing, China), and 16S rDNAs were amplified with primers 27F (5′AGAGTTTGATCMTGGCTCAG3′) and 1492R (5′TACGGYTACCTTGTTACGACTT3′) primers (Loaces et al., 2011).

### Soil Analysis

The soil was collected with ectomycorrhizal roots from sites where truffle fruiting occurred, including Hebei, Liaoning, Yunnan, and Heilongjiang Provinces. The samples were dried and used to determine the soil properties. Analyses were performed with standard protocols at the Institute of Plant Nutrition and Resource, Beijing Academy of Agriculture and Forestry Sciences and also followed the methods of Benucci et al. (2016). Organic matter (OM), total nitrogen (N), assimilable phosphorus (P), exchangeable potassium (K), magnesium (Mg), calcium (Ca), soil granularity and pH were measured. Significant differences between different sites were tested using one-way ANOVA.

### Characterization of Nitrogen Fixation and Phosphorus-Solubilizing Traits

For the cultured bacteria isolated from *T. indicum*, nitrogen-fixation activity was tested in two ways: whether it can grow on Burk’s N-free medium (Wilson and Knight, 1974; Park et al., 2005), and whether nifH gene can be amplified by PolF/PolR primer set on bacterial DNA (Poly et al., 2001). A single colony of each purified strain was spotted on National Botanical Research Institute’s phosphate growth medium (NBRIP) plates, and observed for halo/zone formation (Ramachandran et al., 2007; Dastager et al., 2011). Furthermore, the nitrogen-fixation activity of those bacteria growing on N-free medium was verified with an ARA in the State Key Laboratory of Agricultural Microbiology of Huazhong Agricultural University according to previously described methods for legume root nodules (Wych and Rains, 1978; Das and De Kumar, 2018) and combined with a modified method by Barbieri et al. (2010). In brief, the cultured bacteria strains were incubated for 2 h at 28°C in 30 ml glass bottles containing 2 ml of C_2_H_2_. The amount of ethylene was measured using an East & West Analytical Instrument GC 4000A gas chromatograph.

## Results

### The Morphological Characteristics and Phylogenetic Relationship Between the Analyzed Samples of *T. indicum*

**FIGURE 1:**
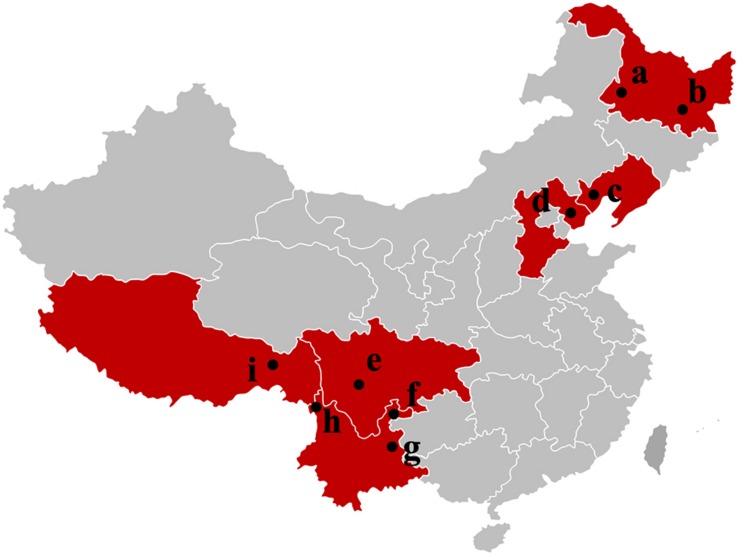
Samples map of *T. indicum* fruiting bodies collected in this study. a: Qiqihar city, Heilongjiang Province; b: Mudanjiang city, Heilongjiang Province; c: Chaoyang city, Liaoning Province; d: Zunhua city, Hebei Province; e: Liangshan Yi Autonomous Prefecture, Sichuan Province; f: Zhaotong city, Yunnan Province; g: Qujing city, Yunnan Province; h: Gongshan Derung and Nu Autonomous county, Yunnan Province; i: Linzhi city, Tibet.

**FIGURE 2:**
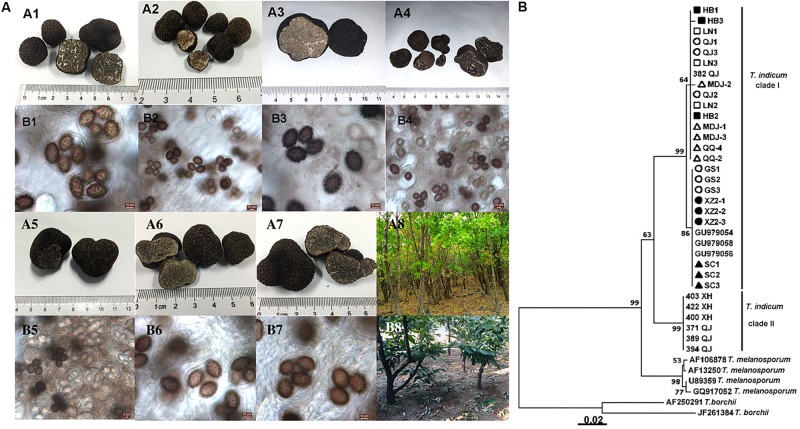
Morphological characteristics of ascocarps and ascospores of *T. indicum* **(A)** and phylogenetic analysis of all samples from different regions of China based on ITS sequence using the Neighbor-joining analysis **(B)**. Morphological characters of ascocarps (black with conspicuous warts) (A1–A7) and ascospores (brown to dark, ornamented with spine) (B1–B7), respectively. Samples collected from Gongshan Derung and Nu Autonomous County, Yunnan Province (A1,B1); Zunhua County, Hebei Province (A2,B2); Liaoning Province (A3,B3); Mudanjiang City, Heilongjiang Province (A4,B4); Qiqihar City, Heilongjiang Province (A5,B5), Qujing County, Yunnan Province (A6,B6); Tibet (A7,B7). (A8,B8) showed the host tree: *Quercus mongolica* and *Castanea mollissima*, respectively. **(B)** The neighbor-joining tree based on ITS sequences of *T. indicum* from different regions. △HB1-HB3: samples from Hebei Province; ■LN1-LN3: samples from Liaoning Province; ⚫QJ1-QJ3: samples from Qujing county, Yunnan Province; ▲MDJ1-MDJ3: samples from Mudanjiang, Heilongjiang Province; ○GS1-GS3: samples from Gongshan, Yunnan Province; □XZ2-1-XZ2-3: samples from Linzhi city, Tibet; ▼QQ2, QQ4: samples from Qiqihar, Heilongjiang Province; ▽SC1-3: samples from Liangshan, Sichuan Province.

*Tuber indicum* is a typical Chinese black truffle species with great morphological variation and wide distribution. In this study, we collected 120 fruiting bodies of *T. indicum* from nine sites across Northeastern and Southwestern China, including Zhaotong County, Qujing City, Gongshan County (Yunnan Province), Liangshan County (Sichuan Province), Linzhi County (Tibet), Zunhua City (Hebei Province), Mudanjiang City and Qiqihar City (Heilongjiang Province) and Chaoyang City (Liaoning Province) (Figure 1). The morphological characteristics and phylogenetic relationships of the representative samples of *T. indicum* analyzed in our study are shown in Figure 2. All samples collected from the nine sites had similar morphological features, namely, black ascomata with conspicuous warts, ellipsoidal ascospores with sparse spine. Based on ITS sequencing and phylogenetic analysis, all samples analyzed belong to the same clade with high bootstrap values (100%), indicating the relatively consistent genetic background of all samples.

### Diversity of *T. indicum* Bacterial Communities From Different Regions

**FIGURE 3:**
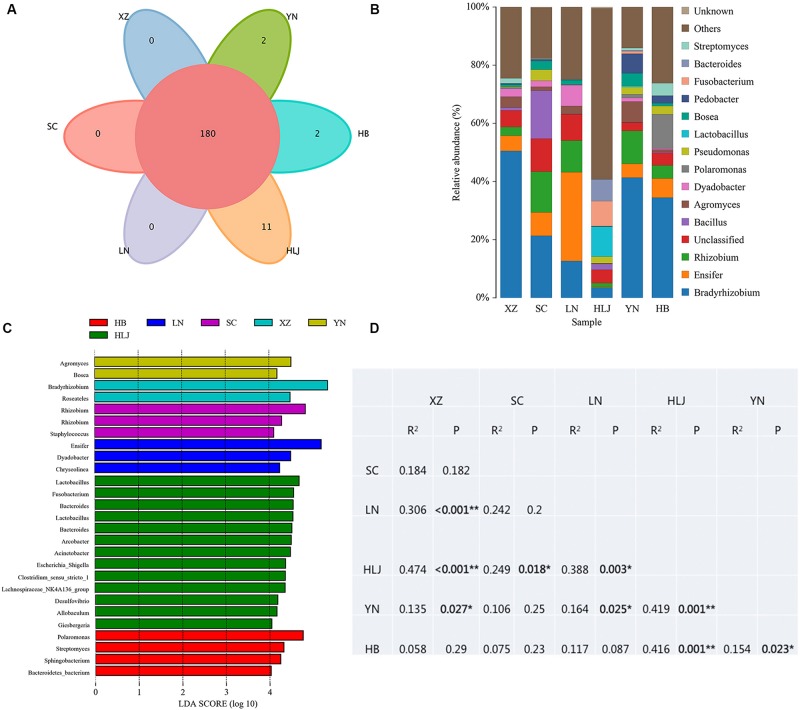
Effect of geographical location on bacterial composition of fruiting bodies of *T. indicum.* **(A)** Venn diagram showed the shared bacteria community across geographic locations at the genus level; **(B)** Bacteria relative abundance at the genus level across geographic regions; **(C)** Differentially abundant genus in fruiting bodies of *T. indicum* depending on geographical regions, as inferred by the LEfSe algorithm. The threshold for the logarithmic discriminant analysis (LDA) score was 4, with a *P* value of <0.05. **(D)** PERMANOVA analysis shows that the bacterial communities are significantly different in the fruiting bodies of *T. indicum* from different regions of China. HB, Hebei Province; LN, Liaoning Province; SC, Sichuan Province; XZ, Tibet; YN, Yunnan Province.

High throughput sequencing of the V3 + V4 region of 16S rDNA was carried out on 54 fruiting bodies of *T. indicum* (3–9 replicates each site) with the Illumina HiSeq platform. A total of 3981692 reads were obtained from the representative fruiting bodies of *T. indicum* from the nine sites. Although it did not capture the entire community, the rare faction curve and Shannon curve indicated that the sequencing depth basically covered the bacterial species of the samples (Supplementary Figure S1). After quality filtering, 3450293 sequences were clustered into 695 OTUs. The results of OTUs taxonomy and relative abundances are listed in Table 1. Comparatively, the bacterial communities of *T. indicum* ascocarps from different regions showed that the number of OTUs was highest in the Hebei region (310 ± 11) and the lowest in the Liaoning region (274 ± 18), but no significant differences were observed between the samples from different geographic locations. The Shannon index of the bacterial community was highest in samples from Yunnan Province (3.5039 ± 0.0418) and significantly different from other regions. All of the OTUs were distributed across 20 phyla, 153 families, and 307 genera. Among the 307 genera, 180 genera were shared between samples from different regions, no specific genus was distributed in the Sichuan, Tibet and Liaoning regions, and only 2 genera were specific in samples from Yunnan and Hebei Province, and 11 genera exhibited geographical enrichment in Heilongjiang samples, including *Barnesiella, Allobaculum, Nitrosomonas, Plasticicumulans, Rheinheimera*, and other unclassified bacteria (Figure 3A).

**TABLE 1:** Richness estimates and diversity indices of the bacterial communities in fruiting bodies of *T. indicum* produced from different regions of China.

**Collecting site**	**OTUs**	**Ace index**	**Chao1 index**	**Simpson index**	**Shannon index**
Hebei Province	310 ± 11a	335.86 ± 16.13a	334.61 ± 44.06a	0.0738 ± 0.0047b	2.1343 ± 0.2101b
Heilongjiang Province	303 ± 42a	337.93 ± 42.18a	340.78 ± 13.76a	0.188 ± 0.0517ab	2.3701 ± 0.0995b
Liaoning Province	274 ± 18a	346.47 ± 27.61a	346.47 ± 29.20a	0.2095 ± 0.0986ab	2.5056 ± 0.1561b
Tibet	298 ± 27a	354.86 ± 14.31a	347.60 ± 16.24a	0.2736 ± 0.0442a	2.5266 ± 0.3569b
Sichuan Province	306 ± 22a	366.23 ± 10.37a	371.83 ± 16.20a	0.2818 ± 0.0381a	2.6606 ± 0.3052ab

*Different letters a, b represent significantly difference in the number of OTUs and diversity index between regions.*

### Impact of Geographical Location on Bacteria Composition of *T. indicum* Fruiting Bodies

The result of OTUs taxonomy at the phylum level showed that the relative abundance of Proteobacteria was the highest (36.90–80.79%) in all samples of *T. indicum* ascocarps from different geographic regions, followed by Firmicutes (0.04–37.20%), Bacteroidetes (4.44–14.85%), and Actinobacteria (1.82–10.36%). However, the richness of dominant bacteria was different in *T. indicum* ascocarps across different regions. For example, the relative abundance of Fusobacteria (8.64%) was significantly higher and Proteobacteria (36.90%) and Actinobacteria (1.82%) were lower in samples from Heilongjiang Province compared to those in other regions. Firmicutes was more abundant in samples from Heilongjiang (37.20%) and Sichuan (19.30%) than in other regions, and the difference was significant (*P* < 0.05).

The analysis of OTUs taxonomy at the genus level found that *Bradyrhizobium* (2.66–47.66%) was the most abundant genus in the bacterial communities of *T. indicum* fruiting bodies from different regions, followed by *Ensifer* (0.13–30.03%), *Rhizobium* (1.08–13.89%), *Bacillus* (1.21–16.87%), and *Agromyces* (0.83–7.05%) (Figure 3B). The 10 most observed genera in the fruiting bodies of *T. indicum* from 6 different regions demonstrated that *Bradyrhizobium*, *Lactobacillus*, *Ensifer*, *Rhizobium, Agromyces, Pseudomonas, Streptomyces, Pedobacter, Acinetobacter, Bosea*, and *Pedobacter* are significantly enriched in *T. indicum* fruiting bodies from different geographic regions (Table 2). LEfSe showed significant differences in bacterial groups between regions (Figure 3C). *Agromyces* and *Bosea* were more abundant in Yunnan samples, *Bradyrhizobium* and *Bosea* were more prevalent in Tibet samples, *Rhizobium* and *Staphylococcus* were more enriched in Sichuan samples, *Ensifer*, *Dyadobacter*, and *Chryseolinea* were more abundant in Liaoning samples, *Fusobacterium, Lactobacillus, Bacteroides* and *Acinetobacter* were more enriched in Heilongjiang samples, and *Polaromonas, Streptomyces* and *Sphingobacterium* were more prevalent in Hebei samples. Moreover, the effects of geographic location on the bacterial composition of *T. indicum* ascocarps were analyzed using permutational multivariate analysis of variance (PERMANOVA), and the result reflected that the bacterial communities of *Tuber* fruiting bodies were significantly different across geographical locations (*P* = 0.005) (Figure 3D).

**TABLE 2:** List of the ten most observed bacterial genera associated with *T. indicum* fruiting bodies from different regions.

**Heilongjiang Province**	**Liaoning Province**	**Yunnan Province**	**Sichuan Province**	**Tibet**	**Hebei Province**
**Lactobacillus (9.57%)**	**Ensifer (30.03%)**	**Bradyrhizobium (40.43%)**	**Bradyrhizobium (21.07%)**	**Bradyrhizobium (47.66%)**	Bradyrhizobium (33.4%)
Bacteroides (6.91%)	Bradyrhizobium (l2.86%)	**Rhizobium (11.4S%)**	Bacillus (16.87%)	Ensifer (5.27%)	**Polaromonas (11.8S%)**
Arcobacter (6.78%)	**Rhizobium (11.04%)**	Agromyces (7.05%)	**Rhizobium (13.89%)**	**Agromyces (4.12%)**	Ensifer (6.81%)
Fusobacterium (6.51%)	Dyadobacter (7.28%)	Pedobacter (6.62%)	Ensifer (8.03%)	**Rhizobium (3.37%)**	**Rhizobium (4.82%)**
Acinetobacter (6.44%)	**Agromyces (2.82%)**	Ensifer (4.84%)	Pseudomonas (3.78%)	Dyadobacter (2.96%)	**Streptomyces (4.25%)**
Pseudomonas (3.27%)	Bosea (1.56%)	Bosea (4.02%)	Bosea (2.96%)	**Streptomyces (1.72%)**	Pseudomonas (2.90%)
Bradyrhizobium (2.66%)	Polaromonas (0.15%)	Pseudomonas (2.78%)	Dyadobacter (2.09%)	Bacillus (1.21%)	Pedobacter (2.18%)
Bacillus (1.52%)	Streptomyces (0.13%)	Polaromonas (l.25%)	**Agromyces (1.30%)**	Bosea (0.79%)	Bosea (l.08%)
Rhizobium (1.08%)	Pedobacter (0.11%)	Dyadobacter (l.23%)	Acinetobacter (0.61%)	Pedobacter (0.42%)	Agromyces (0.83%)
Ensifer (0.13%)	Pseudomonas (0.03%)	Fusobacterium (0.95%)	Pedobacter (0.47%)	Polaromonas (0.26%)	Acinetobacter (0.49%)

*Average percentages are given into brackets. Each value of three biological repilicates. Genera which were found to be significantly more present in one region than another based on one-way ANOVA are listed in bold letters.*

**FIGURE 4:**
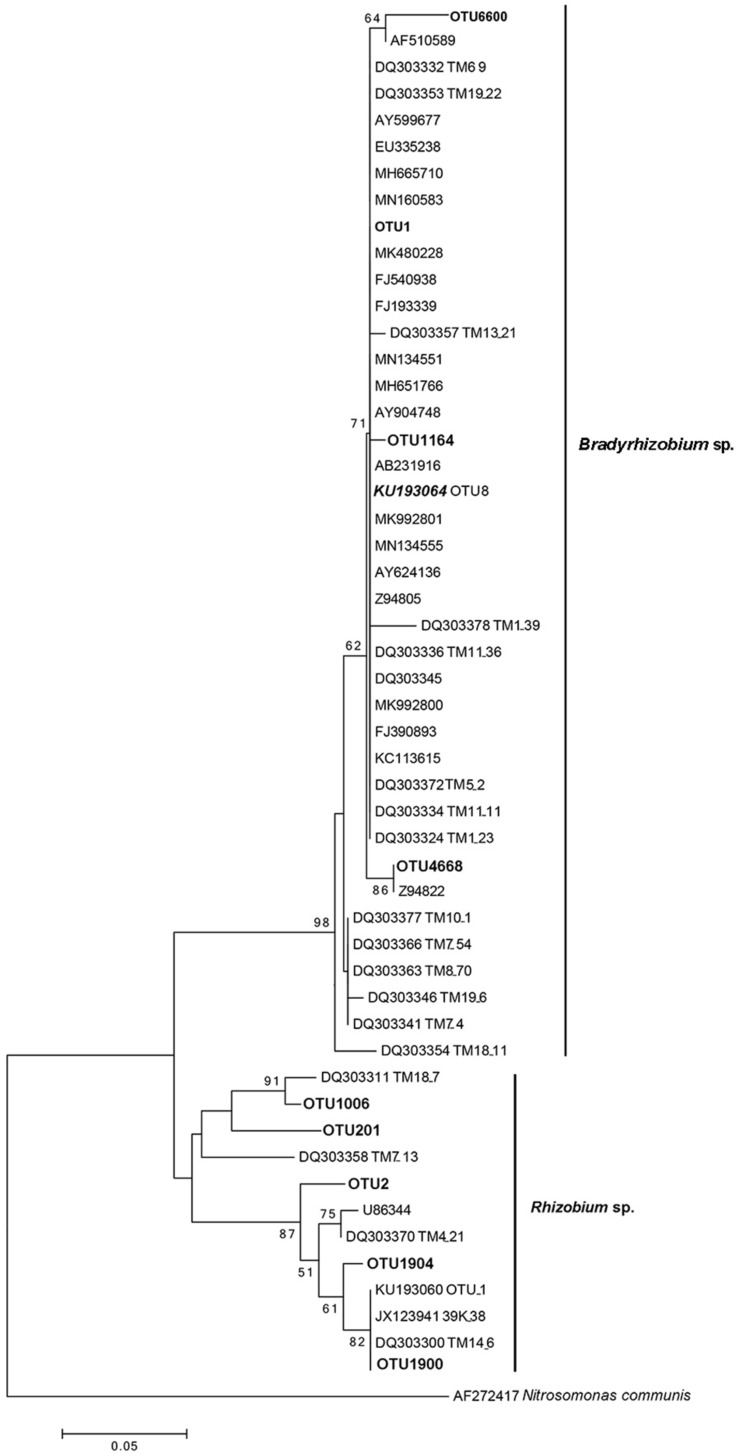
Neighbor-joining phylogenetic tree for 16S rDNA V3 + V4 region sequences of *Bradyrhizobium* spp. and *Rhizobium* spp. from *T. indicum* fruiting bodies. The number at each branch node are the bootstrap numbers from 1000 replicates. OTU1 (dominant taxa), OTU1164, OTU4668 and OTU6600 in bold, belonging to *Bradyrhizobium* and OTU2, OTU201, OTU1006, OTU 1900, and OTU 1904 belonging to *Rhizobium* in this study. KU193064 (OTU8) was regarded as marker *Bradyrhizobium* and specific present in *Tuber* species (Benucci and Bonito, 2016) and sequences of the accession number marked “DQ” were from *T. magnatum* (Barbieri et al., 2007).

Similar to the previous studies, we also confirmed that *Bradyrhizobium* spp. were dominant bacteria in the FBs of *T. indicum* regardless of geographic origin. To compare the phylogenetic relationship between the *Bradyrhizobium* 16S rDNA sequences generated in our study and those published by other authors (Benucci and Bonito, 2016), which were found in the *Tuber* ascomata on several different continents, we conducted a phylogenetic analysis, and the result suggested that the *Bradyrhizobium* 16S rDNA sequences detected in our study (OTU1) were 100% identical to those previously published (KU193064 OTU8), implying that the single *Bradyrhizobium* species is a specific microbe marker in true truffle microbiomes and their potential for tight symbiotic interactions (Figure 4).

Soil properties of the five sites from the four provinces were measured including organic matter (OM), total nitrogen (N), assimilable phosphorus (P), exchangeable potassium (K), calcium (Ca), magnesium (Mg), pH and soil granularity. The results showed that there were significant differences in the contents of Ca and Mg in the Heilongjiang samples but were not significant differences in the contents of K and pH between sites (Table 3). The highest contents of total nitrogen (5.53 ± 0.81), organic matter (113.52 ± 16.44) and effective phosphorus (31.9 ± 10.90) were observed in the Heilongjiang region. The content of changeable magnesium in Hebei (1.71 ± 0.10) was significantly lower than that in other sites (2.37–3.14). Redundancy analysis (RDA) of the correlation between microbial composition and soil property showed that *Bradyrhizobium, Rhizobium, Ensifer*, and *Agromyces* had a positive correlation with soil granularity (0.25 mm ≤ x < 2.00 mm and 0.002 mm ≤ x < 0.02 mm), *Lactobacillus* and *Bacteroides* had a positive correlation with the contents of N, Ca, K, Mg, and P and *Acinetobacter* and *Pseudomonas* displayed a positive correlation with the pH value (Supplementary Figure S2). In summary, bacterial compositions of *T. indicum* fruiting bodies were different from different geographical locations, and the soil parameters are possibly important contributors to the differences in the microbiome.

**TABLE 3:** Differences in soil characteristics of *T. indicum* among geographic sites after one-way ANOVA analysis.

**Geographic regions**	**N (g/Kg)**	**OM (g/Kg)**	**P (g/Kg)**	**K (g/Kg)**	**Ca (g/Kg)**	**Mg (g/Kg)**
Hebei Province	1.455 ± 0.11b	23.6167 ± 1.74b	18.9333 ± 5.13a	0.2763 ± 0.04a	7.4167 ± 0.30b	1.705 ± 0.10^c^
Yunnan Province	3.2 ± 0.18b	8.17ab	7.8 ± 1.61a	0.158 ± 0.03a	10.1667 ± 0.12b	3.1433 ± 0.07^a^
Heilongjiang Province	5.5533 ± 0.81a	113.5167 ± 16.44a	31.9 ± 10.90a	1.2967 ± 0.08a	12.6167 ± 0.33a	2.7817 ± 0.23^ab^
Liaoning Province	1.1033 ± 0.02b	105.7 ± 76.16ab	6.0333 ± 3.18a	1.0467 ± 0.03a	13.0667 ± 0.18a	2.37 ± 0.18^b^

**Geographic regions**			**Soil granularity**			**pH**

	0.25 mm ∼ 2.00 mm(%)	0.05 mm ∼ 0.25 mm(%)	0.02 mm ∼ 0.05 mm(%)	0.002 mm ∼ 0.02 mm(%)	< 0.002 mm(%)	
Hebei Province	46.18 ± 1.08a	13.6067 ± 1.03a	10.3333 ± 0.80b	16 ± 1.03c	13.88 ± 0b	7.2483 ± 0.19^b^
Yunnan Province	19.5833 ± 0.74b	9.2033 ± 0.94b	10.6667 ± 0.67b	33.3333 ± 0.67a	27.2133 ± 1.33b	7.0467 ± 0.17^b^
Heilongjiang Province	18.8933 ± 1.75b	14.2267 ± 1.18a	23 ± 2.67a	20 ± 1.46b	23.88 ± 1.26b	7.2983 ± 0.10^ab^
Liaoning Province	7.97 ± 0.62c	9.4833 ± 0.99b	20 ± 1.15a	28.6667 ± 0.67a	33.88 ± 0b	7.8 ± 0.09^a^

*Different letters a, b, c represents significantly difference in quantity of the soil parameters between regions.*

### Difference in the Bacterial Community Among Ascocarps, Ectomycorrhizae and Soil of *T. indicum*

**FIGURE 5:**
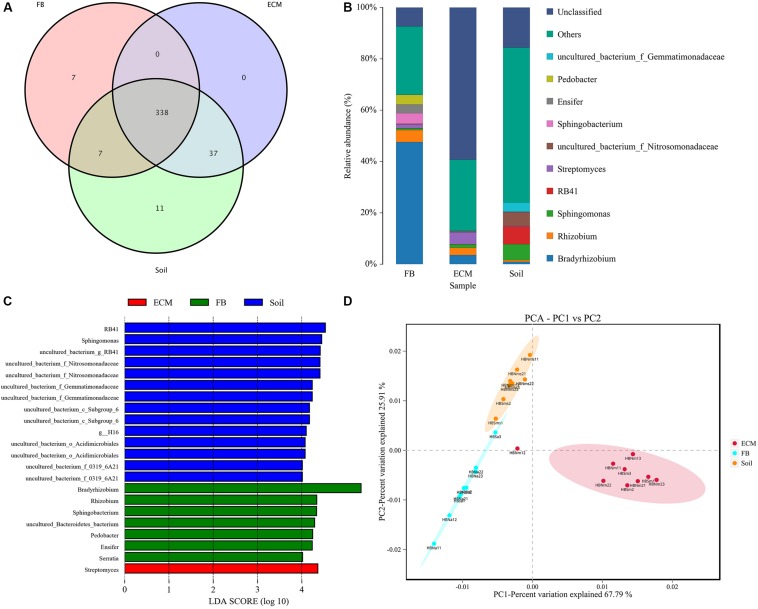
Effect of different compartments (Soil, ECM, and FB) on bacterial composition of *T. indicum*. **(A)** Venn diagram showed the shared bacterial community across three compartments at the genus level; **(B)** Bacterial relative abundance at the genus level across three compartments; **(C)** Differentially abundant genus in different compartments of *T. indicum* inferred by the LEfSe algorithm. The threshold for the logarithmic discriminant analysis (LDA) score was 4, with a *P* value of <0.05. **(D)** PCA analysis shows relationships in bacterial composition among samples belonging to different compartments and three compartments display significant different. ECM, Ectomycorrhiza; FB, Fruiting bodies.

In addition to exploring the bacterial composition of fruiting bodies of *T. indicum* from different geographical regions, we also determined the difference in bacterial composition colonizing the fruiting body (FB), ectomycorrhizae (ECM) and ectomycorrhizosphere soil of *T. indicum* on samples collected in September 2018 at the same time, from the same sites and from the same host tree (*Castanea mollissima*). Based on 16S rDNA V3 + V4 region sequencing, we sequenced 24 samples comprising 6 soils, 9 ectomycorrhizaes (more than 300 root tips) and 9 FBs samples, generating 2419871 high-quality reads. After filtering, we obtained 64693 clean tags and assigned 1503 OTUs, and the ectomycorrhizosphere soil samples contained more OTUs than the ECM and FB of samples *T. indicum*. The highest Shannon index value was observed in ectomycorrhizosphere soil (6.08 ± 0.03), followed by ectomycorrhizae (2.85 ± 0.19) and ascocarps (2.55 ± 0.16), indicating that the bacterial diversity was significantly different among the soil, fruiting bodies and ectomycorrhizae of *T. indicum*. All of the OTUs were distributed across 21 phyla, 218 families and 384 genera. A lot of 338 of 384 genera were shared between different compartments, and no specific genus was detected in the ECM. The numbers of specific genera in the FBs and soils were 7 and 11, respectively (Figure 5A).

The OTUs taxonomy results of three compartments of *T. indicum* at the phylum level showed that *T. indicum* FBs harbored a core bacterial community that was mainly composed of Proteobacteria (76.95%) and Bacteroidetes (12.90%); the ECM of *T. indicum* was dominated by Proteobacteria (30.17%) and Actinobacteria (12.25%), while soil samples contained mainly Proteobacteria (44.26%) and Acidobacteria (26.04%). At the genus level, all OTUs from three compartments were assigned to 384 genera. *Bradyrhizobium* (46.22%) and *Rhizobium* (5.29%) were significantly enriched in the FBs, *Streptomyces* (4.65%), *Bradyrhizobium* (3.60%) and *Sphingomonas* (1.35%) were prevalent in the ECM, while RB41 (7.97%), *Sphingomonas* (6.26%) and uncultured bacteria were dominated in the soil (Figure 5B). The result of LEfSe analysis showed significant differences in bacterial groups among the three compartments (Figure 5C). RB41, *Sphingomonas* and members of Nitrosomonadaceae and Gemmatimonadaceae were more enriched in the soil samples, *Streptomyces* were more abundant in the ECM samples, and *Bradyrhizobium*, *Rhizobium, Pedobacter, Ensifer*, and *Serratia* were more prevalent in the FB samples. PCA among three main compartments (FB, ECM, and soil) also revealed that the bacterial communities in soil were separated from the other two compartments according to the first axis, which explained 67.79% of the variability, and from those of the ectomycorrhzae according to the second axis, which explained 25.91% of the variability (Figure 5D).

### The Cultural Bacterial Communities

A total of 60 bacterial colonies were isolated from ethanol-sterilized fruiting bodies of *T. indicum* by the tissue culture method. The 60 colonies were gathered from 26 different strains according to morphological and molecular characteristics. The 26 strains were divided into 4 phyla and 24 genera based on 16S rDNA sequencing (Supplementary Figure S3). Among them, the largest phylum was Proteobacteria (43 strains, 71.67%), including *Pseudomonas* (9 strains), *Alcaligenes* (5 strains), *Ensifer* (4 strains), *Inquilinus* (3 strains), *Mesorhizobium* (3 strains), *Roseomonas* (3 strains), *Variovorax* (3 strains), *Rhizobium* (2 strains), *Acinetobacter* (2 strains), *Enterobacter* sp. (2 strains), *Stenotrophomonas* (2 strains) and five other genera with only one strain. Moreover, the members of Actinobacteria were also dominant in the cultural bacteria from *T. indicum* fruiting bodies (21.67%), including *Microbacterium* (5 strains), *Arthrobacter* (5 strains) and three other genera. A total of three strains of *Elizabethkingia* (1 strain) and *Sphingobacterium* (2 strains) belonging to Bacteroidetes consisted of 5%, and *Bacillus* (1 strain) and *Paenibacillus* (1 strain) belonging to Firmicutes consisted of 3.33% (Table 4). All of these bacteria members were also found dominant in the fruiting bodies of *T. indicum* using high-throughput sequencing, confirming their presence in fruiting bodies. All bacterial strains were deposited in the Mycological lab of the Institute of Medicinal Plant Development, Chinese Academy of Medical Sciences.

**TABLE 4:** Identification information about 26 strains cultural bacteria from fruiting bodies of *T. indicum.*

**No. of isolated strain**	**Reference species in NCBI**	**Class/Phylum**	**Accession Number**	**Identification%**
T1	*Mesorhizobium* sp.	α*-*Proteobacteria	KM083696	98
T2	*Klebsiella variicola*	γ-Proteobacteria	MH290480	100
T3	*Ochrobactrum* sp.	α*-*Proteobacteria	MK517588	100
T4	*Acinetobacter calcoaceticus*	γ-Proteobacteria	LT984792	100
T5	*Arthrobacter nicotinovorans*	Actinobacteria	FN908773	99
T6	*Bosea* sp.	α*-*Proteobacteria	LCI 33722	100
T7	*Microbacterium esteraromaticum*	Actinobacteria	MG719571	100
T8	*Bacillus amyloliquefaciens*	Firmicutes	MK063798	100
T9	*Alcaligenes faecalis*	β-Proteobacteria	KX023244	100
T10	*Agrobacterium tumefaciens*	α-Proteobacteria	MH429810	100
T11	*Chryseobacterium indologenes*	Actinobacteria	KR002424	100
T12	*Variovorax ginsengisoli*	β-Proteobacteria	AB649024	99
T13	*Brevundimonas* sp.	α-Proteobacteria	KU257689	100
T14	*Kocuria rosea*	Actinobacteria	MK532258	100
T15	*Roseomonas* sp.	α-Proteobacteria	KC753761	100
T16	*Microbacterium flavescens*	Actinobacteria	EU714363	100
T17	*Ensifer adhaerens*	α-Proteobacteria	EF442029	100
T18	*Enterobacter ludwigii*	γ-Proteobacteria	JF690979	100
T20	*Pseudomonas* sp.	γ-Proteobacteria	KJ733977	100
T21	*Stenotrophomonas* sp.	γ-Proteobacteria	KT034431	100
T24	*Elizabethkingia* sp.	Bacteroidetes	CPO16377	100
T25	*Inquilinus limosus*	α-Proteobacteria	LN995702	100
T26	*Microbacterium resistens*	Actinobacteria	MK517595	100
T27	*Rhizobium* sp.	α-Proteobacteria	MF621574	100
T28	*Sphingobacterium* sp.	Bacteroidetes	KM077439	100
T29	*Variovorax paradoxus*	β-Proteobacteria	FJ527675	97

### Nitrogen-Fixing Activity and Phosphorus-Solubilizing Traits

Out of the 26 tested strains, 13 strains (50%) were found to grow in Burk’s N-free medium including T1-T6, T8-T9, T15, T17-T18, T20, and T27, which belong to Proteobacteria and Firmicutes, respectively (Supplementary Figure S4). The nitrogen fixation activity of one strain (T20) was verified by enzymatic ARA test (Supplementary Figure S5). Eight strains harbored the nifH gene, which was determined by PCR amplification using specific primers, including strains T1, T2, T4, T8, T13, T18, T20, and T21 (Supplementary Figure S6). Only one strain, T4 (*Acinetobacter calcoaceticus*) belonging to γ-Proteobacteria showed the highest phosphate solubilization activity. Strain T4 exhibited two different activities: phosphate solubilization and nitrogen fixation bioactivity.

## Discussion

Unlike other edible mushroom, truffles are produced and developed underground during the entire lifecycle. In recent years, soil microbes associated with mycorrhizae and fruiting bodies of several commercial species of *Tuber* have been investigated based on culture-dependent and high-throughput sequencing, including *T. borchii, T. melanosporum, T. magnatum*, *T. aestivum*, and *T. brumale* (Gazzanelli et al., 1999; Barbieri et al., 2005; Buzzini et al., 2005; Antony-Babu et al., 2014; Deveau et al., 2016). These results indicated that a considerable number of bacteria colonize truffle fruiting bodies, while *Bradyrhizobium*, *Variovorax*, *Pedobacter*, and *Sphingobacterium* are usually enriched in fruiting bodies of *T. melanosporum* (Antony-Babu et al., 2014). Similar to *T. melanosporum*, *T. indicum* was enrichment in a core set of bacterial taxa despite of the significant difference in composition of the six different geographical regions investigated. *Bradyrhizobium, Ensifer, Rhizobium, Bacillus, Agromyces, Dyadobacter*, and *Polaromonas* were dominant in *T. indicum* fruiting bodies. The enriched core bacteria in *T. indicum* fruiting bodies overlap with the reported core set for *T. melanosporum* (Antony-Babu et al., 2014), *T. aestivum* (Gryndler et al., 2013), *T. magnatum* (Barbieri et al., 2007), *T. panzhihuaensis* (Wan and Liu, 2014), *T. lyonii* and *T. oregonense* (Benucci et al., 2016), which placed in different clades in the phylogenetic tree, raising the possibility that a number of bacterial genera (e.g., *Bradyrhizobium*, *Ensifer*, *Sphingobacterium*, and *Rhizobium*) have had a long association with truffle, at least since the divergence of truffle from other fungal taxa in approximately 271 million years ago, implying some core bacterial communities most likely co-evolution with truffle (Jeandroz et al., 2008). Moreover, *Pseudomonas* has been reported to be enriched in white truffle *T. borchii* and *T. magnatum* (ascocarp surface white and no conspicuous warts) with high frequency (Bedini et al., 1999; Barbieri et al., 2007), but in black truffle, *T. melanosporum* is rarely observed (Mello et al., 2013; Antony-Babu et al., 2014). Similarly, a very low proportion of *Pseudomonas* was detected in *T. indicum* in our study with a culture-independent method.

Interestingly, the most representative *Bradyrhizobium* sp. (very closely related to *B. elkanii*) (OTU1) in *T. indicum* FBs in our study are identical to those described in other *Tuber* species (KU193064 OTU8) by Benucci and Bonito (2016), which were found to be dominant specifically in the genus *Tuber* irrespective of geographic origin but not in other truffle genera. Our results verified that *Bradyrhizobium* (OTU1) is consistently present in all *T. indicum* FBs collected from various locations although the proportion is different in each tested samples. Moreover, *Bradyrhizobium* and *Rhizobium* are important root-associated microbes known for their nitrogen-fixing capability (Hara et al., 2019). In this study, we detected that the *T. indicum* FBs have nitrogen-fixing activities and suspected that the activity is probably related to the presence of *Bradyrhizobium* spp. as previously reported in *T. magnatum* (Barbieri et al., 2010). Thus, the potential effects of nitrogen-fixing bacteria on the development of truffle in cultivation should be considered.

Deng et al. (2018) surveyed the bacterial community of *T. indicum* fruiting bodies from Yunnan and Sichuan Province using 454 pyrophosphate sequencing and found 220 OTUs belonging to 7 phyla and 43 genera. In this study, we explored more abundant bacterial taxa (20 phyla and 307 genera) in the fruiting bodies of *T. indicum* samples collected from different geographical regions, including samples collected from Yunnan and Sichuan Provinces. In addition to, *Flavobacterium, Agromyces*, *Microbacterium*, *Ensifer*, *Sphingopyxis*, and *Variovorax* are dominant in *T. indicum* fruiting bodies according to previous reports. In the present study, *Bradyrhizobium* (2.66–47.66%), *Rhizobium* (1.08–13.89%), and *Bacillus* (1.21–16.87%) etc., are also dominant in *T. indicum* fruiting bodies. Our survey substantially increases the number of bacterial taxa enriched in *T. indicum* fruiting bodies and emphasizes the important effect of the extensive sampling on bacterial diversity, as mentioned by Splivallo et al. (2019). Furthermore, PERMANOVA analysis also supported the difference in bacterial abundance in different samples from various geographic locations. The correlation analysis of RDA between soil propriety and microbial composition indicated that the enrichment of the bacterial community in the truffle was affected by soil physical and chemical characters, which probably contribute to the geographical difference of the microbes in the *T. indicum* fruiting bodies.

The microbial community are also different between different compartments of *T. indicum*, such as soil, ectomycorrhizae and fruiting bodies. *Bradyrhizobium* appeared to be more prevalent in the FBs (47.6%) than in the ECM (3.53%) or the soil (less than 1%). This result is consistent with previous result in other truffle species such as *T. melanosporum* (Antony-Babu et al., 2014) and *T. aestivum* (Gryndler et al., 2013). Similar to *T. melanosporum*, *Rhizobium*, and *Streptomyces* are dominant in the microbial composition of *T. indicum* ECM. A possible explanation is that FBs of truffle are site for accumulation of carbohydrate, protein and lipid, while ECM is a place where small molecules substances transfer and exchange; and the different metabolites and functions present in the ECM and FB possibly resulted in the difference in the microbial communities in the two parts. Overall, different compartments of *T. indicum* are enriched with different bacteria, indicating that *T. indicum* has selectivity for bacterial colonization during their development.

Plant growth-promoting rhizobacteria (PGPR) are acknowledged as new and promising biotechnology tools in agriculture field for enhancing nursery plant production, the growth of mycorrhizal plants, and sporocarp yield in recent years (Bhattacharyya and Jha, 2012; Navarroródenas et al., 2016). Their effect on the truffle mycorrhizal synthesis and mycelium growth were also reported (Dominguez et al., 2012; Gryndler et al., 2013; Navarroródenas et al., 2016). In our study, we isolated a total of 60 bacterial colonies from fruiting bodies of *T. indicum* and represented 26 different strains based on morphological traits and molecular sequences. Molecular identification using the 16S rDNA sequence divided the strains into 4 phyla and 24 genera, of which the largest were *Pseudomonas* of the Proteobacteria phylum (15% of the isolated strains), although a low abundance was detected in *T. indicum* FBs by 16S rDNA high-throughput assay. None of the members of *Bradyrhizobium* sp., the prevalent bacteria in the FBs of *T. indicum* using high-throughput sequencing, were isolated and cultured in artificial medium, confirming the complementary advantage of methodologies for deciphering the bacterial communities of truffle.

The bioactivity analysis displayed that 13 strains of 26 different taxa can grow in Burk’s N-free medium, implying the potential ability of nitrogen fixation, including *Mesorhizobium* sp., *Klebsiella variicola, Acinetobacter calcoaceticus*, *Arthrobacter nicotinovorans, Roseomonas* sp., *Ensifer adhaerens, Pseudomonas* sp., and *Bacillus amyloliquefaciens* etc., *Pseudomonas* and *Bacillus* have been the most reported as PGPR bacteria (Islam et al., 2014; Navarroródenas et al., 2016). Only one strain, *Acinetobacter calcoaceticus*, displayed the ability to solubilize phosphate and fix nitrogen in this study. These bacteria may be potential candidates for the formation and development of ectomycorrhizae and fruiting bodies of truffle, and the exact ecological function of these bacteria will be further validated in the next step by inoculating experiments on truffle mycorrhizal seedlings.

In conclusion, our results provide a comprehensive view of bacteria communities of *T. indicum* FBs from different geographic areas. We confirmed the dominance of *Bradyrhizobium* sp. in FBs of *T. indicum* and verified its close phylogenetic relationship with those in other *Tuber* species, regardless the geological location even across the continent. The core bacteria most likely coevolved with truffle and play an important role in the nutrition requirement and adaptation to the environment during truffle development. The diversity of bacterial communities is similar in FBs of *T. indicum* but relative abundance and richness are different from different geographical origins. The bacterial composition colonized in mycorrhizosphere soil, ectomycorrhizae, and fruiting bodies of *T. indicum* are significantly different. Partial representative bacterial strains in fruiting bodies of *T. indicum*, which were detected using a culture-independent method, were also isolated and cultured, confirming their occurrence during the development of fruiting bodies of *T. indicum* to some extent. Some cultural bacteria strains exhibited the potential abilities of nitrogen fixation and inorganic phosphorus solubilization, suspecting that these bacteria play important function in truffle seedling growth, fruiting body development and even truffle evolution process. Overall, this study provides important data for understanding the interaction between bacterial communities and fruiting bodies of *T. indicum* and provides useful microbe resource for *T. indicum* cultivation in future.

## Data Availability Statement

The datasets generated for this study can be found in the NCBI database with GenBank accession numbers MK346212–MK346219.

## Author Contributions

JC and S-XG designed the experiments. J-ML and Y-JT performed the experiments. PQ, P-GL, Y-MX, and YL collected the samples. JC and J-ML analyzed the data, and contributed to the text writing.

## Conflict of Interest

The authors declare that the research was conducted in the absence of any commercial or financial relationships that could be construed as a potential conflict of interest.
