# Supplementary material for: Chinese Black Truffle-Associated Bacterial Communities of Tuber indicum From Different Geographical Regions With Nitrogen Fixing Bioactivity
Source: Front Microbiol. 2019 Nov 5;10:2515. doi: 10.3389/fmicb.2019.02515 (PMC6848067; doi:10.3389/fmicb.2019.02515)
Supplement: Supplementary file 1 [file Data_Sheet_1.docx]

Supplementary Material

Chinese Black Truffle-Associated Bacterial Communities of *Tuber indicum* From Different Geographical Regions With Nitrogen Fixing Bioactivity

Juan Chen^1*^, Jia-Mei Li^1^, Yan-Jing Tang^1^, Yong-Mei Xing^1^, Peng Qiao^2^, Yang Li^1^, Pei-Gui Liu^3^, Shun-Xing Guo^1^*


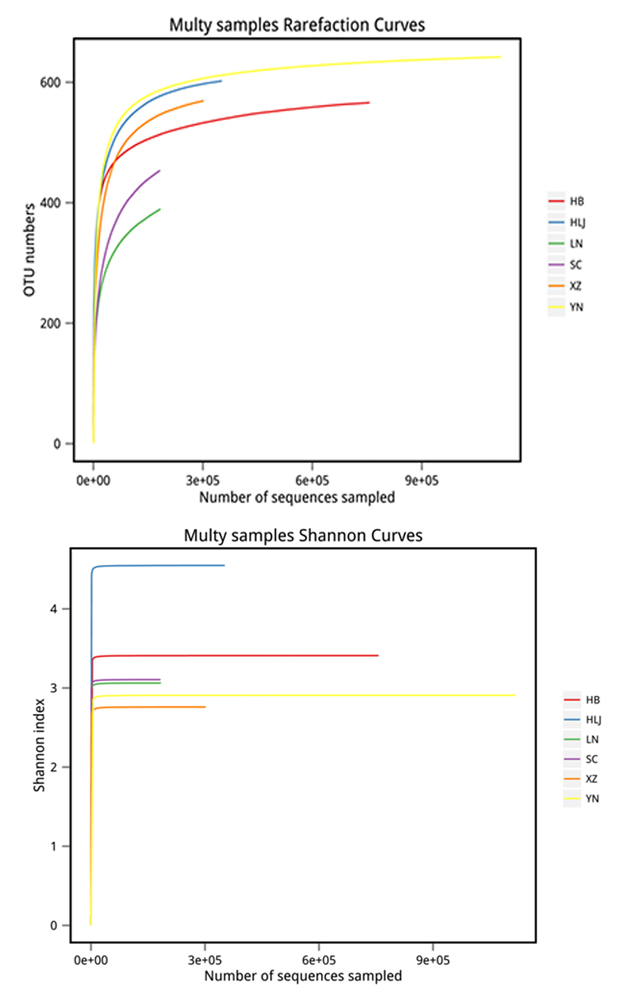


**Figure S1.** **Rarefaction curve and diversity of Shannon curve.** The curves reach saturation for all samples studied, meaning the sequencing depth is enough for diversity analysis.


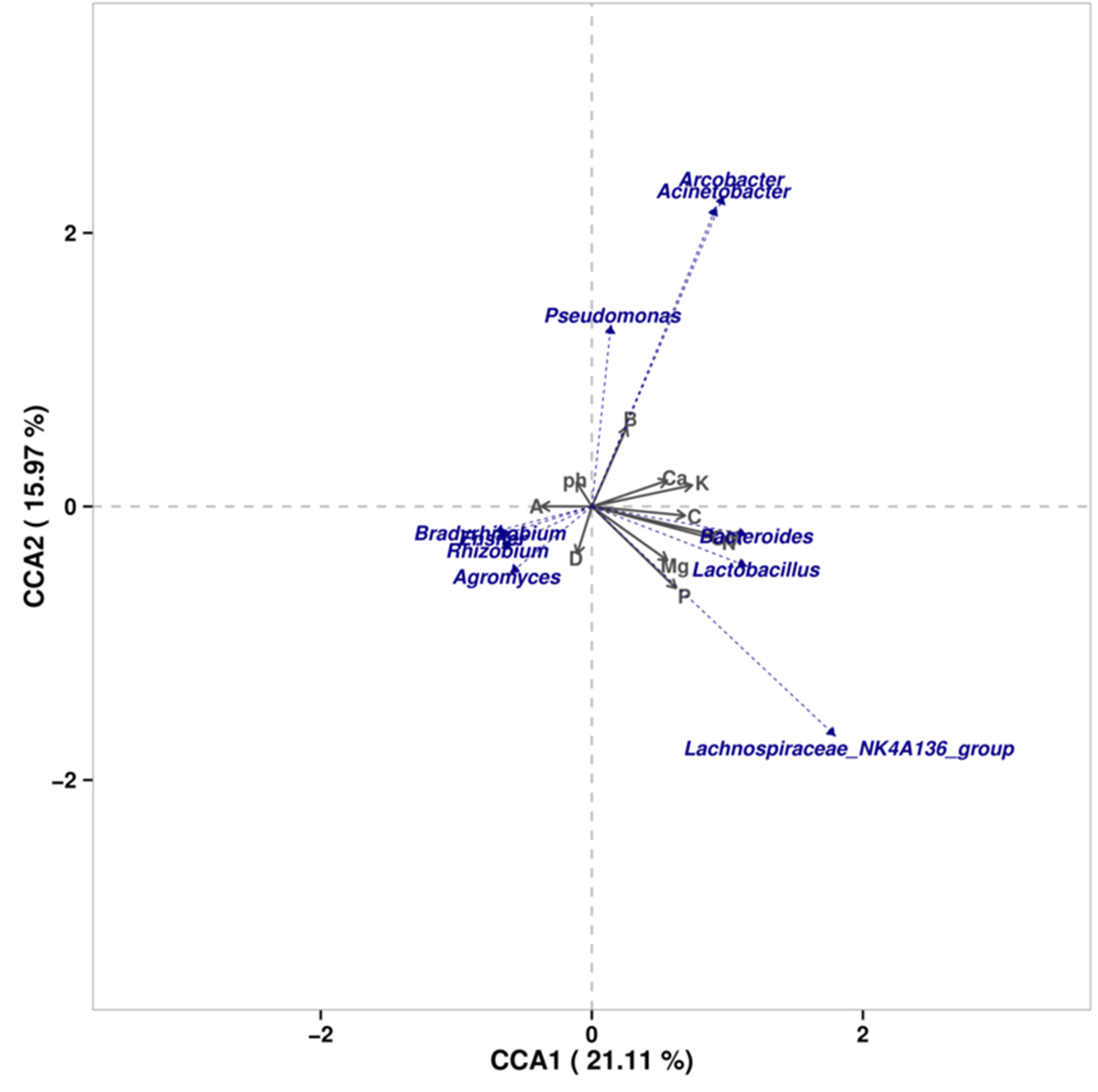


**Figure S2**. **The effect of soil property on soil bacterial composition using the RDA (Redundancy analysis).** The result showed that the correlations between bacterial composition and soil property in spite of the low CCA value. *Bradyrhizobium, Rhizobium, Ensifer* and *Agromyces* have positive correlation with soil granularity (0.25mm≤x<2.00mm and 0.002mm≤x<0.02mm), and *Lactobacillus* and *Bacteroides* has positive correlation with the content of N, Ca, K, Mg, P and *Acinetobacter, Pseudomonas* display positive correlative with pH value.





**Figure S3. The neighbor-joining tree conducted based on 16S rDNA sequence using the MEGA 6.0 software to identify the cultural bacteria (T1-T29) isolated from fruiting bodies of *T. indicum*.** The number along branch represents the bootstrap value above 50%.


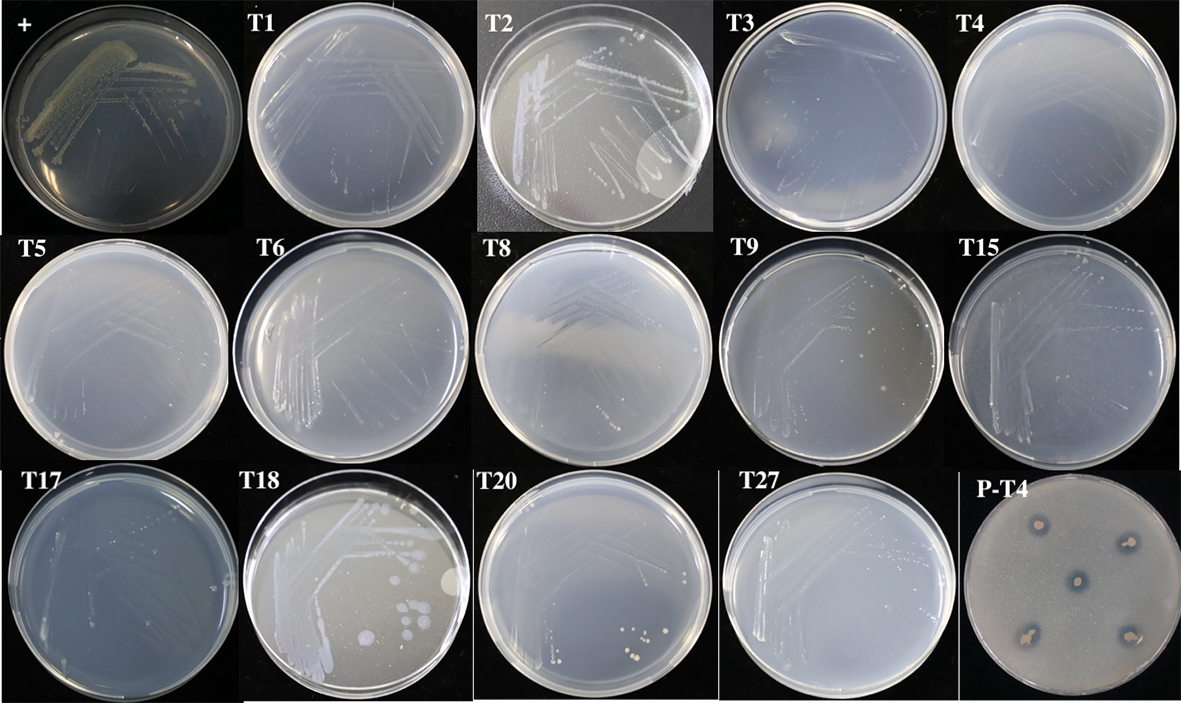


**Figure S4. Detection of nitrogen-fixing and phosphate-solubilizing activitive of cultural bacteria isolated from *T. indicum*.** Positive control (+) : *Azotobacter chroococcum*. Nitrogen-fixing activity: strains T1-T6，T8-T9，T15，T17-18，T20，T27 can grow in Burk’s nitrogen free medium; Phosphate-solubilizing activity: strain P-T4 can grow in the medium, indicating its potential solubilize phosphate activity.


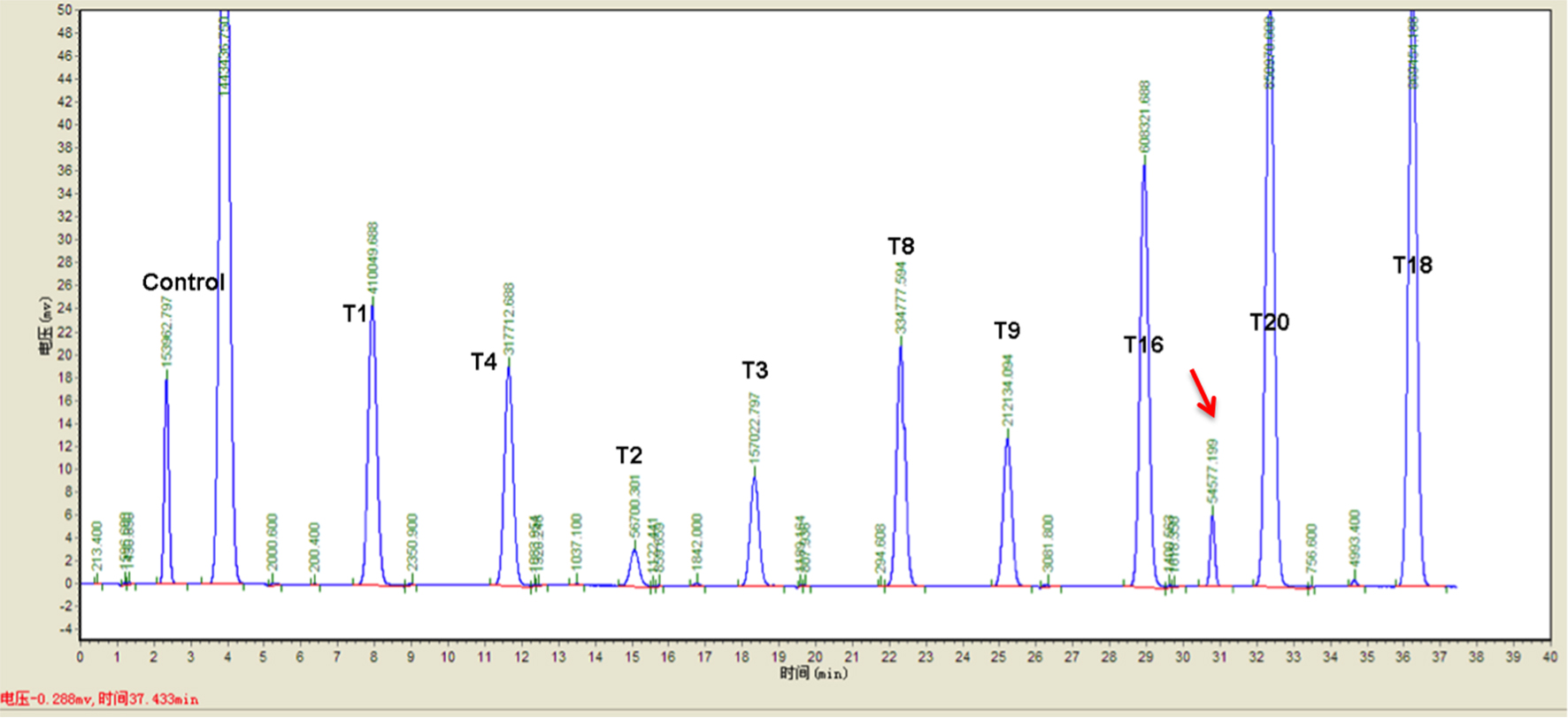


**Figure S5. Nitrogen-fixing activity of strains (T1-T4，T8-T9，T16，T18，T20) was tested by acetylene reduction assay.** Only one strain T20 (*Pseudomonas* sp.) was detected with nitrogen-fixing activity (marked in red).


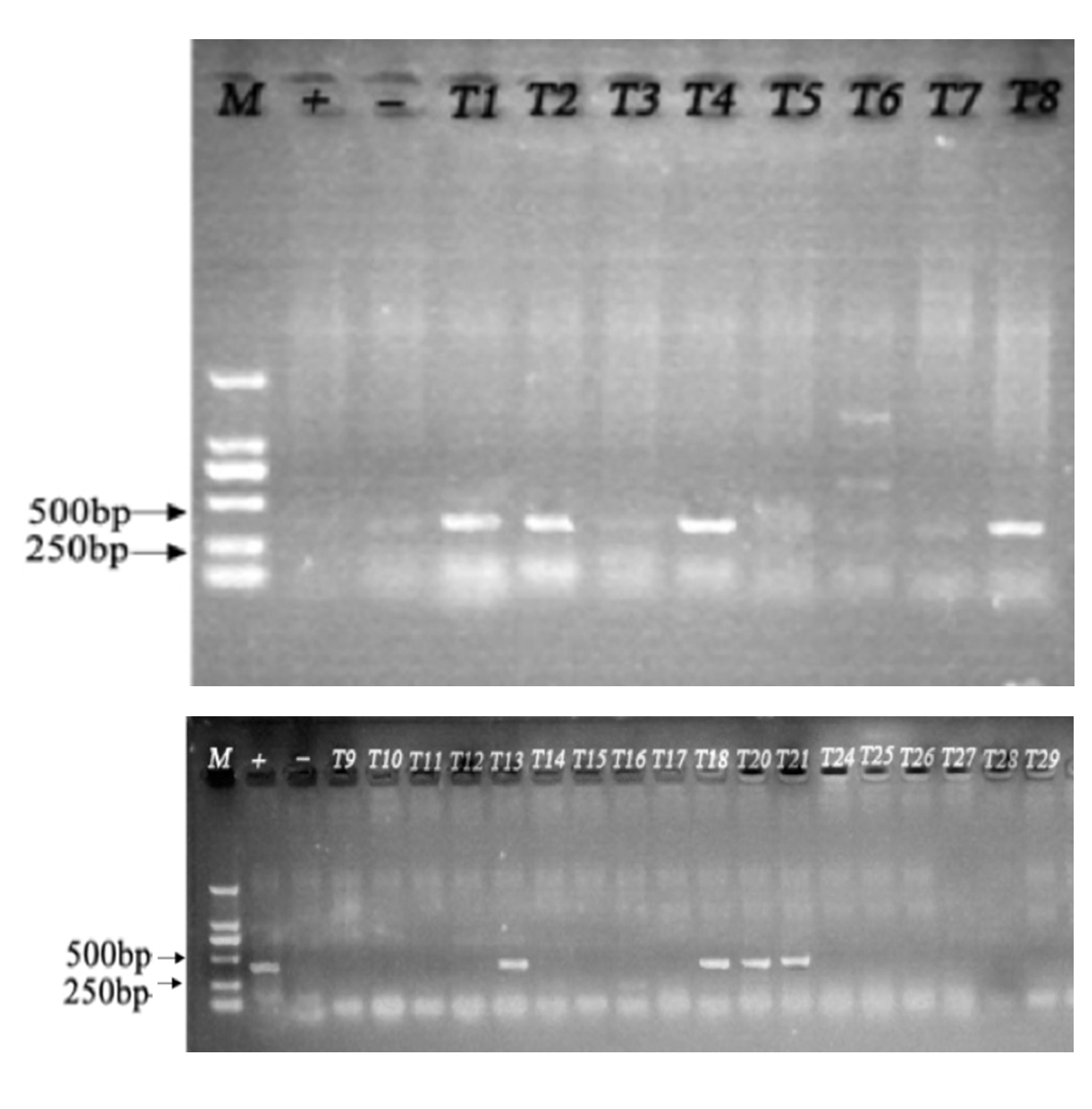


**Figure S6. The PCR amplification tested the cultural bacteria with nif gene.** Eight strains (T1, T2, T4, T8, T13, T18, T20, T21) were identified with nif gene products, indicating potential nitrogen fixation activity.
